# Increased Prevalence of Autoimmune Rheumatologic Diseases in Patients With Primary Hyperparathyroidism

**DOI:** 10.7759/cureus.46906

**Published:** 2023-10-12

**Authors:** Elif Güneş, Mutlu Güneş

**Affiliations:** 1 Department of Endocrinology, Metabolism and Diabetes, Health Sciences University, Bursa State Hospital, Bursa, TUR; 2 Department of Endocrinology, Metabolism and Diabetes, Health Sciences University, Bursa Yuksek Ihtisas Training and Research Hospital, Bursa, TUR

**Keywords:** systemic lupus erythematosus, parathormone, dickkopf-related protein 1, ankylosing spondylitis, behçet’s disease, rheumatoid arthritis, autoimmune rheumatologic diseases, primary hyperparathyroidism

## Abstract

Background

Parathyroid hormone (PTH) and Dickkopf-related protein 1 (DKK-1) have been mentioned together at the intersection of autoimmune rheumatologic diseases (ARDs) and osteoimmunology. However, few studies have evaluated the association between primary hyperparathyroidism (PHPT) and ARDs.

Methodology

This retrospective study included 225 PHPT patients and 386 patients with thyroid nodules as a control group. The electronic hospital records of all patients were screened going back nine years for the presence of ARDs. Patients who were diagnosed at least three months ago, had complete serologic tests, and were continuing with rheumatologic follow-up were included.

Results

The prevalence of ARDs in the PHPT group was 9.77% (22/225), while the prevalence of ARDs in the CG was 1.04% (4/386, p < 0.001). The prevalence of rheumatoid arthritis in the PHPT group was 4.4% (10/225), ankylosing spondylitis 3.1% (7/225), systemic lupus erythematosus 0.88% (2/225), Behçet’s disease 0.88% (2/225), and mixed connective tissue disease 0.44% (1/225). Of the 22 patients with ARDs, 21 (95.45%) were diagnosed before they were diagnosed with PHPT, and the median time from diagnosis with ARD to the onset of PHPT was 36 months (interquartile range = 61.5). Logistic regression analysis showed a positive correlation between the duration of PHPT and ARDs (odds ratio (OR) = 1.06; 95% confidence interval (CI) = 1.02-1.09, p < 0.001) and a negative correlation between ARDs and calcium levels (OR = 0.26; 95% CI = 0.09-0.79, p = 0.018).

Conclusions

The prevalence of ARDs increased in PHPT patients and PHPT accompanying ARDs developed after rheumatologic disease. ARDs with PHPT are cases with a prolonged duration of PHPT and mildly elevated calcium, probably preceded by parathyroid hyperplasia. Therefore, the factors that cause ARDs may trigger a process that leads to mild PHPT.

## Introduction

Parathyroid hormone (PTH), a peptide, is secreted by the parathyroid glands. The narrow serum calcium (Ca) level, essential for many metabolic processes, is regulated by the Ca-sensing receptor that regulates PTH release, with PTH acting through its G protein-coupled receptors (GPCRs) [1]. The PTH1 receptor (PTH1R) is expressed in bone and regulates Ca ion homeostasis through its activation [1]. PTH affects a wide range of specialized bone cells, including osteoblasts and stromal cells. Its effect on osteoclasts is indirect and mediated through osteoblasts. Through its receptors located on osteoblasts, PTH has several effects that directly promote bone formation, but the most important physiological effect is the stimulation of osteoclast differentiation and development. PTH plays an important role in the renal conversion of 25-hydroxyvitamin D (25(OH)D) to its active form, 1,25-dihydroxyvitamin D (1,25(OH)D). The metabolism of 25(OH)D, one of the main determinants of PTH and its variability, may be impaired in inflammatory diseases [2].

Primary hyperparathyroidism (PHPT) is a common endocrine disorder characterized by excessive PTH secretion from one or more parathyroid glands, with resulting high PTH levels accompanied by hypercalcemia [3]. The cause is a solitary parathyroid adenoma in 80% of cases, hyperplasia in 10-15% of cases, multiple adenomas in 5% of cases, and parathyroid carcinoma in <1% of cases [4]. Once considered an endocrine disorder characterized by severe hypercalcemia and skeletal and renal complications, the incidence of primary PHPT has increased as serum Ca measurements have become routine. With this increase, symptomatic PHPT cases have decreased, asymptomatic PHPT cases have started to be seen and even a third variant, defined as normocalcemic PHPT, has been identified [3,4]. Currently, PHPT is the third most common endocrine disorder after diabetes and thyroid disorder, with a prevalence of 2-7 per 1,000 adults [5].

In PHPT, the disease process in both asymptomatic and normocalcemic cases may result in bone resorption and increased fracture risk with the effect of PTH, independent of Ca level [3]. Despite being long recognized as an endocrine disorder, many uncertainties remain in the pathophysiology of PHPT. The nuclear factor-kB receptor activator (RANK)/nuclear factor-kB ligand-receptor activator (RANKL)/osteoprotegerin (OPG) system, fibroblast growth factor-23 (FGF-23), sclerostin, Klotho, and Dickkopf-related protein 1 (DKK-1) systems have been investigated in PHPT patients [6].

The Wnt pathway is involved in bone formation. DKK-1 negatively regulates canonical Wnt signaling by binding to and antagonizing Wnt lipoprotein receptor-related proteins (LRP) 5-6 coreceptors (LRPcR) [7]. PTH is a major determinant of DKK-1 effects and plays a crucial role in bone development. PTH suppresses the expression of DKK-1 in osteoblasts [7]. Recently, the close association of PHPT with DKK-1 has been discussed [6,7].

Patients with symptomatic PHPT may present with arthralgias and even erosive synovitis, which may mimic rheumatoid arthritis [8]. However, the recent view is that “overt rheumatologic manifestations are mainly a historical phenomenon and are not part of the clinical spectrum of modern disease” [9]. In rheumatoid arthritis (RA) patients, hyperparathyroidism has been associated with cortical erosions, low bone formation, and secondary osteoporosis through overexpression of DKK-1 [10]. PTH has been shown to be one of the main determinants of DKK-1 serum levels in ankylosing spondylitis (AS) [11]. Dysregulation of wingless Wnt signaling has been detected in systemic lupus erythematosus (SLE) patients, and DKK-1 has been proposed as an independent biomarker for bone erosion and even lupus nephritis [12]. Most recently, 136 rheumatologic diseases in 136 PHPT patients referred to rheumatology specialists were identified. The most common autoimmune rheumatologic disease (ARD) was RA (n = 16, 11.7%), followed by primary Sjogren’s syndrome (pSS) (n = 12, 8.8%) and SLE (n = 5, 3.6%) [13].

Although evidence has been presented indicating that PTH may play a role in the pathogenesis of ARDs and may be a biomarker for ARDs, few studies have evaluated the prevalence of rheumatologic diseases and the relationship between them in patients with PHPT. In this study, we aimed to determine the prevalence of ARDs in PHPT patients and discuss this relationship in light of the available data.

## Materials and methods

This retrospective cohort study included 225 PHPT patients admitted to a tertiary endocrinology, diabetes, and metabolic diseases clinic between September 2020 and August 2023 and 386 age-and-sex-matched patients with thyroid nodules as a control group. The study was approved by the Ethics Committee of the University of Health Sciences, Bursa Faculty of Medicine, Bursa State Hospital (E-13012450-514.99-2222846164) and was conducted in accordance with the principles of the Declaration of Helsinki.

Inclusion criteria

The inclusion criteria were male and female patients over 18 years of age diagnosed with symptomatic or asymptomatic PHPT. Age- and gender-matched patients who were followed for euthyroid thyroid nodules were included as a control group.

Exclusion criteria

Patients with normocalcemic PHPT, tertiary and secondary hyperparathyroidism, familial hypocalciuric hypercalcemia, and PHPT due to familial multiple endocrine neoplasms were excluded.

Study design and work plan

The baseline clinical characteristics of patients were obtained from hospital records. These included the following: time of onset of PHPT, gender, age, rheumatologic diseases, time of onset of rheumatologic diseases, presence of comorbid diseases, laboratory findings (albumin-corrected serum levels of calcium, phosphate, PTH, 25(OH)D, creatinine and 24-hour urine calcium, and uric acid), PHPT complications (osteoporosis, nephrolithiasis), and radiologic findings. The diagnosis and differential diagnosis of PHPT were made for all patients according to current guidelines [4].

In the electronic hospital records of the patients, diagnosed ARDs accompanying PHPT were collected. Similarly, in the euthyroid thyroid nodule group (the control group), accompanying diagnosed ARDs were also collected. In both groups, only patients with a positive autoantibody profile and no definitively diagnosed ARDs were excluded from the statistical analysis.

The PTH reference range was 15-65 ng/L, and the Ca reference range was 8.8-10.2 mg/dL. A serum 25(OH)D level <30 µg/L was accepted as the cut-off point for deficiency [4]. Hypercalcemia was categorized as mild when the Ca levels were between 10.2 and 12.0 mg/dL, moderate when the levels were between 12.0 and 14.0 mg/dL, and severe when the levels were higher than 14.0 mg/dL [4]. Hypophosphatemia was defined as a confirmed serum level below 2.5 mg/dL. The hyperuricemia threshold was accepted as 6.0 mg/dL. The clinical diagnosis of osteoporosis is based on the presence of fragility fracture or a T-score ≤-2.5 standard deviations (SDs) in any region based on bone mineral density (BMD) measurement by dual-energy X-ray absorptiometry (DEXA) [4]. Renal involvement is defined as positive if the patient has a history of kidney stones or if a diagnosis of calculi or calcinosis is made on ultrasound or computed tomography [4].

Measurement of biochemical and hormonal parameters

Serum intact PTH levels were measured using an immunoassay method (Cobas c801, Roche Diagnostics Corporation, Indianapolis, IN, USA). Serum 25(OH)D levels were measured using the high total electrochemiluminescence method (Cobas e801, Roche Diagnostics Corporation, Indianapolis, IN, USA). Total serum Ca, P, and albumin levels were measured using calorimetric methods (Cobas e702, Roche Diagnostics Corporation, Indianapolis, IN, USA). The glomerular filtration rate (GFR) was calculated using the following Chronic Kidney Disease Epidemiology Collaboration creatinine formula:

141 × min (Serum Cr(sCr)/κ, 1)^α^ × max(sCr/κ, 1)^-1.209^ × 0.993^age^ × 1.018 × 1.159

where sCr is measured as mg/dL, κ is 0.7 for women and 0.9 for men, α is -0.329 for women and -0.411 for men, min is the minimum of sCr/κ or 1 and max is the maximum of SCr/κ or 1.

Statistical analysis

Preliminary tests were conducted to determine the distribution of the variables. The normally distributed results of the Kolmogorov-Smirnov test were expressed as mean ± SD; results that were not normally distributed were expressed as median and interquartile range (IQR) of 25-75 percentiles. Continuous variables with non-normal distributions using the Mann-Whitney U test and normal distributions were analyzed using the t-test for unpaired samples. Unpaired continuous data correlations were analyzed using Pearson’s correlation test, and Spearman’s tests were used to determine the correlation between the unpaired binary data. Binary logistic regression analyses were applied to determine whether the correlated data were independent factors. The level of statistical significance was set at p-values <0.05. All calculations were performed using SPSS version 20 (IBM Corp., Armonk, NY, USA).

## Results

The PHPT group consisted of 225 patients (female (F)/male (M) = 187/38), and the control group consisted of 386 patients (F/M = 308/78). The mean age of the PHPT population was 56.6 ± 12.5 years, and the mean age of the control group was 55.6 ± 9.6 years. The median duration of PHPT was 18 months (IQR = 31). Demographic data for the patient group and the control group are provided in Table 1.

**Table 1:** Demographic and laboratory characteristics of the PHPT group and the thyroid nodule group (control group). *: As it does not show a normal distribution, median and an interquartile range (IQR) of 25–75 percentiles are given. PHPT = primary hyperparathyroidism; BMI = body mass index; Cr = creatinine; GFR = glomerular filtration rate; Ca = calcium; P = phosphorus; PTH = parathyroid hormone; 25(OH)D = 25-hydroxyvitamin D; DEXA = dual-energy X-ray absorptiometry; FT = femur total; L1-4 = lumbar vertebrae 1–4; ARDs = autoimmune rheumatologic diseases

Variable	PHPT group (n = 225)	Thyroid nodule group (n = 386)	P-value
Age (year)	56.6 ± 12.5	55.6 ± 9.6	0.25
Female/male	187/38	308/78	0.31
BMI (kg/m^2^)	30.0 (IQR = 5.5)*	27.8 (IQR = 5.5)*	0.030
Cr (mg/dL)	0.70 (IQR = 0.30)*	0.70 (IQR = 0.27)*	0.80
GFR (mL/minute)	93.0 (IQR = 29.0)*	93.0 (IQR = 21.0)*	0.58
Ca (mg/dL)	11.5 (IQR = 1.0)*	9.2 (IQR = 0.7)*	<0.001
P (mg/dL)	2.45 (IQR = 0.77)*	3.40 (IQR = 0.60)*	<0.001
PTH (ng/L)	152.0 (IQR = 163.0)*	37.0 (IQR = 15.3)*	<0.001
Uric acid (mg/dL)	5.00 (IQR = 1.70)*	4.50 (IQR = 1.80)*	<0.001
25(OH)D (µg/L)	16.0 (IQR = 12.1)*	20.0 (IQR = 14.0)*	0.016
TSH (μIU/mL)	1.45 (IQR = 1.49)*	1.55 (IQR = 1.60)*	0.48
DEXA			
L1–4 (T-score)	-1.81 ± 1.43		
L1–4 (Gr/cm^2^)	0.989 ± 0.183		
FT (T score)	-1.59 ± 1.03		
FT (Gr/cm^2^)	0.820 ± 0.148		
ARDs, number (%)	22/225 (9.78%)	4/386 (1.04%)	<0.001

**Figure 1:**
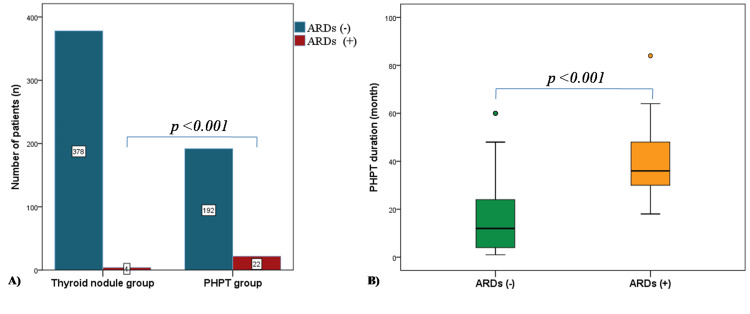
The frequency of autoimmune rheumatologic diseases (ARDs) in the primary hyperparathyroidism (PHPT) group and the thyroid nodule group (A), the relationship between the duration of PHPT and ARDs (B).

The observed prevalence of ARDs in the PHPT group was 9.77% (22/225), while the observed prevalence of ARDs in the control group was 1.04% (4/386) (Figure 1, Panel A).

Autoimmune rheumatologic diseases

The 22 patients with PHPT and ARDs had a median age of 55.6 ± 10.5 years at inclusion. The median duration of ARD was 80 months (IQR = 60), while the median duration of PHPT associated with ARDs was 39.0 months (IQR = 27.0). Of the 22 patients with ARDs, 21 (95.45%) were diagnosed before they were diagnosed with PHPT, and the median time from ARD to the onset of PHPT was 36 months (IQR = 61.5). The prevalence of ARDs between groups is presented in Table 2.

**Table 2:** Distribution of ARDs between groups. autoimmune rheumatologic diseases; PHPT= primary hyperparathyroidism.

ARDs	PHPT group (n = 225)	Thyroid nodule group (n = 386)
Rheumatoid arthritis	4.4% (10)	0.51% (2)
Ankylosing spondylitis	3.1% (7)	0.25% (1)
Behçet’s disease	0.88% (2)	
Systemic lupus erythematosus	0.88% (2)	
Mixed connective tissue disease	0.44% (1)	
Primary Sjogren’s syndrome		0.25% (1)

The demographic and laboratory characteristics of PHPT patients with and without concomitant ARDs are provided in Table 3.

**Table 3:** Demographic and laboratory characteristics of patients with PHPT with and without ARDs. *: As it does not show a normal distribution, the median and an interquartile range (IQR) of 25–75 percentiles are given. ARDs = autoimmune rheumatologic diseases; BMI = body mass index; Cr = creatinine; GFR = glomerular filtration rate; Ca = calcium; P = phosphorus; PTH = parathyroid hormone; U Ca = 24-hour urinary calcium; 25(OH)D = 25-hydroxyvitamin D; DEXA = dual-energy X-ray absorptiometry; FT = femur total; L1-4 = lumbar vertebrae 1–4; PHPT = primary hyperparathyroidism

Variable	ARDs (+) (n = 22)	ARDs (-) (n = 203)	P-value
Age (year)	55.6 ± 10.5	56.7 ± 12.7	0.69
Female/male	19/3	168/35	0.67
BMI (kg/m^2^)	27.7 (IQR = 4.0)*	27.8 (IQR = 5.8)*	0.97
Cr (mg/dL)	0.73 (IQR = 0.30)*	0.70 (IQR = 0.30)*	0.73
GFR (mL/min)	93.0 (IQR = 31.0)*	90.0 (IQR = 28.5)*	0.49
Ca (mg/dL)	11.20 (IQR = 1.25)*	11.50 (IQR = 1.00)*	0.20
P (mg/dL)	2.60 (IQR = 0.80)*	2.40 (IQR = 0.70)*	0.26
PTH (ng/L)	125.0 (IQR = 106.3)*	157.0 (IQR = 173.0)*	0.16
Uric acid (mg/dL)	5.70 (IQR = 2.90)*	4.90 (IQR = 1.7)*	0.157
U Ca (mg/Day)	242.0 (IQR = 130.3)*	330.0 (IQR = 235.0)*	0.037
25(OH)D (µg/L)	20.5 (IQR = 15.0)*	14.8 (IQR = 12.5)*	0.034
DEXA			
L1–4 (T-score)	-1.94 ± 1.47	-1.77 ± 1.40	0.55
L1–4 (Gr/cm^2^)	1.005 ± 0.146	0.989 ± 0.182	0.70
FT (T score)	-1.79 ± 0.84	-1.50 ± 1.06	0.37
FT (Gr/cm^2^)	0.810 ± 0.143	0.832 ± 0.150	0.68
PHPT duration (month)	36.0 (IQR = 24.0)*	17.7 (IQR = 20.0)*	<0.001

In the PHPT group, the mean duration of PHPT was longer in those with ARDs compared to those without (39.0 months (IQR = 27.0) and 12.0 months (IQR = 21.5), respectively, p < 0.001) (Figure 1, Panel B). While 77.2% (17/22) of PHPT associated with ARDs could not be localized by imaging modalities, only 11.1% (25/225) of those without ARDs could not be localized.

25(OH)D levels were higher in PHPT patients with ARDs compared to those without ARDs, and 24-hour urine Ca levels were lower in PHPT patients with ARDs compared to those without (Table 3).

Correlation analysis and logistic regression analysis

There was a correlation between ARDs and PHPT, 25(OH)D, uric acid, and the duration of PHPT. These correlations are presented in Table 4.

**Table 4:** Bivariate correlation analysis. PTH = parathyroid hormone; Ca = calcium; 25(OH)D = 25-hydroxyvitamin D; ARDs = autoimmune rheumatologic diseases; PHPT = primary hyperparathyroidism; r = correlation coefficient; p = statistical significance value

Variable		Group	Gender	Age	PTH	Ca	25(OH)D	ARDs	Uric acid
Gender	r	0.043							
	p	0.286							
Age	r	0.070	-0.059						
	p	0.085	0.142						
PTH	r	0.752	0.087	-0.058					
	p	<0.001	0.142	0.329					
CA	r	0.575	-0.042	0.110	0.511				
	p	<0.001	0.513	0.085	<0.001				
25(OH)D	r	-0.14	-0.003	0.041	-0.363	-0.212			
	p	0.015	0.926	0.487	<0.001	0.001			
ARDs	r	0.217	0.041	0.010	0.030	0.008	0.131		
	p	<0.001	0.315	0.803	0.615	0.907	0.044		
Uric acid	r	0.194	-0.280	0.216	0.111	0.266	0.119	0.162	
	p	<0.001	<0.001	<0.001	0.113	<0.001	0.118	0.002	
PHPT duration	r	.	0.308	0.045	-0.123	0.064	0.064	0.422	0.064
	p	.	0.638	0.580	0.131	0.438	0.432	<0.001	0.478

One of the main findings of our study that ARDs preceded PHPT was obtained through logistic regression analysis to identify independent factors; the duration of PHPT was found to be an independent factor influencing ARDs, or there was a correlation with the duration of PHPT. In addition, the duration of PHPT was longer in the PHPT group with ARDs (Tables 3, 4). Localization efforts were less successful in PHPT associated with ARDs. Furthermore, PHPT was less severe in the PHPT with ARDs group, based on the fact that in the PHPT group, there was a negative correlation between ARDs and calcium levels (Table 5).

**Table 5:** Determination of independent factors affecting ARDs by logistic regression analysis. ARDs = autoimmune rheumatologic diseases; Ca = calcium; PTH = parathyroid hormone; 25(OH)D = 25-hydroxyvitamin D; PHPT = primary hyperparathyroidism; OR = odds ratio; CI = confidence interval

Dependent Variable	Independent variable	β	Wald Chi-square	P-value	OR	OR (95% CI)	
						Lower	Upper
	Constant	3.73	0.72	0.40	41.6		
Model 1	Age	-0.51	3.03	0.082	0.95	0.90	1.00
	Gender	-0.44	0.25	0.62	0.64	0.11	3.66
ARDs	Ca	-1.35	5.60	0.018	0.26	0.09	0.79
	PTH	0.003	1.66	0.20	1.00	0.99	1.00
	25(OH)D	0.01	0.09	0.77	1.01	0.94	1.09
	Uric acid	0.44	2.97	0.09	1.55	0.94	2.55
	PHPT duration	0.05	12.17	<0.001	1.06	1.02	1.09

## Discussion

In this cohort study, the prevalence of ARDs observed in PHPT patients was 9.77% compared to 1.04% in the control group. Of the patients with ARDs, 95.45% were diagnosed before they were diagnosed with PHPT. This is one of the few studies in the literature to describe the frequency of ARDs in PHPT patients and the relationship between the duration of ARDs and PHPT onset. Our study clearly demonstrated an association between PHPT and various ARDs.

Studies on different ARDs have mentioned PTH and DKK-1 together at the intersection of the etiopathogenesis and the course of rheumatologic diseases and in the context of a new field of research called osteoimmunology [14]. PTH has been shown to induce osteoblast differentiation, mainly through the activation of the canonical Wnt pathway, the primary regulator of bone turnover [15]. Thus, bone formation is achieved through the effects of the Wnt pathway on osteoblast functionality. The Wnt pathway can also inhibit osteoclastic bone resorption [16]. The two main antagonists of this system are sclerostin and DKK-1. In particular, DKK-1 is expressed by both osteocytes and osteoblasts and strongly inhibits Wnt signaling [17]. The most important hormones affecting DKK-1 and sclerostin are PTH and 25(OH)D [18]. DKK1 serum levels are significantly increased in PHPT patients compared with control subjects [18,19]. In addition, the treatment of osteoporosis with teriparatide is associated with increases in serum DKK1 [18]. The physiologic balance between PTH action and established Wnt-pathway inhibitors, with their altered interplay in the presence of hyperparathyroidism, may explain the skeletal involvement in PHPT, which can range from normal bone to severe osteoporosis. Extensive studies are needed to confirm this hypothesis.

Sainaghi et al. [20] found that PTH levels were higher in patients with ARDs than in the control group and that ARDs were independent predictors of high PTH levels. Increased PTH levels have been associated with joint erosion, and it is hypothesized that T lymphocytes promote PTH-induced osteoclastogenesis by increasing the sensitivity of stromal cells to PTH [14]. As shown in both healthy subjects and patients with PHPT, persistent PTH signaling increases the RANKL/RANK pathway activity, negatively correlates with sclerostin, and positively correlates with DKK-1 [14]. DKK-1, one of the main regulators of which is PTH, seems to determine the fate of the arthritic joint, causing the joint remodeling process to progress toward an erosive/destructive phenotype when overexpressed or new bone formation and fibrosis when DKK1 expression is reduced [21]. It may even serve as a biomarker or be used as a potential therapeutic target in rheumatic diseases [21].

RA prevalence rates are approximately 0.5% to 1% of the adult population in developed societies [22]. In our study, the prevalence of RA in PHPT patients was 4.4%. This is much higher than the expected prevalence. In our control group, the prevalence of RA was 0.5%, which is compatible with the literature. In a recent study, the prevalence of PHPT in RA patients was reported to be 2.8% [23]. Among the hormones involved in the pathogenesis of RA, the data point to an important role for PTH. Indeed, a strong association between high PTH levels, aggressive joint erosions, and low BMD, independent of vitamin D, has been documented in RA patients [10]. In RA patients, serum levels of DKK-1 and PTH are significantly elevated despite treatment with glucocorticoids and tumor necrosis factor-alpha inhibitors [24].

In our study, the prevalence of SLE in PHPT patients was 0.88%, which is significantly higher than the general incidence of 0.00125% [22]. PTH1R expression was found to be significantly higher in B-lymphocytes in SLE and pSS patients than in healthy controls, suggesting that PTH may activate B cells in patients with autoimmune diseases [25]. In another study, significantly more DKK-1 protein was detected in both the serum and urine of SLE patients compared to healthy cohorts, and in particular, the concentration of serum DKK-1 was even higher in SLE patients with lupus nephritis than in those without [26]. These results go beyond data that indicate the complex relationship between SLE and PTH and PTH1R expression.

In the present study, the prevalence of AS in PHPT patients was found to be 3.1%, which is significantly higher than the general prevalence of 0.2% [22]. In our control group, the prevalence of AS was 0.25%, which is compatible with the literature. Accumulating evidence gathered to unravel the complex pathogenesis of AS suggests that DKK-1 via the Wnt pathway plays a key role in this disease. Daoussis et al. [27] showed that although AS patients have high DKK-1 levels, DKK-1 is dysfunctional. A meta-analysis that included 1,348 AS patients and 909 healthy controls showed that there was no significant difference in serum DKK-1 levels between AS patients and healthy controls, while patients with high erosion and sclerosis had significantly lower serum DKK-1 levels than the controls [28]. Additional evidence has shown that a reduction in or dysfunction of DKK-1 plays a key role not only in peripheral joint remodeling but also in sacroiliac erosion and fusion, a typical pathologic process of AS [29]. PTH is likely to regulate Wnt signaling in osteoblasts by multiple mechanisms, despite reduced or dysfunctional DKK1. PTH is the main regulator of the RANK/RANKL/OPG system, which controls bone remodeling. RANKL levels have been shown to increase in AS patients compared to controls [30]. Between 18.7% and 62% of AS patients have osteoporosis, and 10% have a collapse fracture [31]. AS is characterized by localized involvement, whereas osteoporosis is diffuse. PTH, via RANKL, may be responsible for diffuse osteoporosis in AS. This needs to be supported by further studies.

BD is rare; its prevalence ranges from 20 to 420/100,000 in Turkey and is 0.64/100,000 in the United Kingdom [32]. In our study, the prevalence of BD in PHPT patients was 0.88%, which is remarkably high. In the largest genetic association study of BD in multiple lineages to date, genotyping was performed on a total of 9,444 patients and controls from seven different populations, and a genetic susceptibility locus for BD was identified in the LNCAROD/DKK-1 intergenic region. LNCARDOD encodes RNA, which acts as an activating regulator of DKK-1 [33].

In fact, PTH is one of the main determinants of serum levels of DKK-1, not only in ARDs but also in many diseases with bone involvement [24]. We would like to emphasize that the effects of PTH in all these rheumatologic pathways occur independently of serum Ca levels and that PTH has its own specific effects. Therefore, the presence of hypercalcemia is not required for these effects. Hypercalcemia is the main metabolic result; when detected incidentally by a blood test, it may actually represent the end point of a long metabolic journey. Therefore, renal and skeletal system damage should be investigated in PHPT patients starting from the first visit. Endocrinologically, perhaps all this can be explained by a third variant of PHPT, normocalcemic PHPT (NPHPT). The international guidelines have recognized NPHPT as a phenotype of PHPT [4,34]. In NPHPT, Ca levels are normal, and PTH levels are slightly lower than in the hypercalcemic form. By definition, ionized Ca, vitamin D levels, urinary Ca excretion, and renal function must be normal to distinguish NPHPT from other variants [35]. NPHPT is associated with end-organ damage, just as PHPT is associated with hypercalcemic cases [35]. Unfortunately, NPHPT cannot be detected in an individual without the measurement of serum PTH. Furthermore, although vitamin D status is very important in autoimmune rheumatic diseases, vitamin D alone is not sufficient to prevent osteoporosis. Despite adequate vitamin D levels, concomitant NPHPT may be one of the mechanisms responsible for osteoporosis. Recently, a significantly higher PTH concentration was described in 105 RA-affected patients compared to 1,020 controls, despite similar plasma vitamin D and Ca concentrations [36]. The lack of suppression of PTH in rheumatologic diseases despite adequate vitamin D replacement supports this hypothesis. Chronically high serum PTH levels, even when not accompanied by high Ca, may contribute to adverse PHPT-related outcomes, such as increased bone porosity and decreased cortical thickness. All PHPT cases in our study group were hypercalcemic patients, but we would like to emphasize that Ca levels were lower in the PHPT group with ARDs compared to those without ARDs as an independent factor in the regression analysis. We know that NPHPT may develop before the development of typical hypercalcaemic PHPT, as a patient series showed that hypercalcemia developed in 0.6-19% of NPHPT patients [3]. In this case, it is not enough to measure only serum Ca. To prevent this situation, PTH measurement should be performed at least once a year. To improve this situation, periodic evaluation of comorbidities in both disease groups should be kept in mind. Perhaps if the NPHPT had been evaluated in the course of rheumatological disease, our results might have been different. This prediction needs to be supported by further studies. Based on these results, studies aiming to clarify the roles of differential PHPT variants in ARDs are needed.

PTH has been proposed as one of the main hormones regulating DKK-1 in inflammatory conditions. The functioning of PTH, which mainly has a regulatory role in this system, may be impaired in inflammatory diseases. These results may suggest that PHPT itself does not cause ARDs. The fact that all but one of the PHPT cases in our study (21/22, 95.45%) developed during the course of rheumatological disease suggests that PHPT may develop as a comorbidity of rheumatic diseases. In a rare study investigating the association of ARDs and PHPT, 32 of 42 (76.2%) patients were diagnosed with ARDs either before or concurrently with their diagnosis with PHPT, while 10 (23.8%) were diagnosed after being diagnosed with PHPT [13]. In the process of ARDs, a mechanism that disrupts the normal functioning of the parathyroids may trigger hyperactivity of the parathyroid glands. Thus, high or inappropriate normal PTH levels begin to contribute to joint/bone damage in ARDs. In our study, we found that 77.2% (17/22) of PHPT cases associated with ARDs could not be localized by imaging methods. The failure to localize possible parathyroid adenoma in PHPT associated with ARDs suggests the presence of multiglandular disease (i.e., hyperplasia) rather than parathyroid adenoma. Fifth International Workshop reports have documented that all localization efforts are less successful in NPHPT compared to hypercalcemic PHPT, and the prevalence of multiglandular disease is approximately two to three times higher in NPHPT than in PHPT [4]. It has been concluded that problems with parathyroid localization in NPHPT may be related to the higher incidence of multiglandular disease [4]. The logistic regression analysis in the present study found that PHPT cases associated with ARDs had a longer PHPT duration. This result is due to the failure to localize these cases and the consequent delay in surgery. Again, in our study, a striking result of the logistic regression analysis was that PHPT patients with ARDs had significantly lower Ca levels. Our knowledge that NPHPT may progress to overt PHPT suggests that these cases may have developed in the background of NPHPT. Further studies are needed to confirm this hypothesis. Interacting mechanisms in ARDs, as in chronic renal failure, seem to stimulate not only PTH but also parathyroid gland hyperplasia. Of course, this information needs to be supported by the prospective studies. Once PHPT develops, it negatively affects the outcomes for rheumatologic patients and increases their comorbidities.

A better understanding of the pathophysiology of this system, which still has many unanswered questions, could be crucial in preventing and treating joint/bone damage and osteoporosis in patients with inflammatory/autoimmune diseases.

The strengths of our study are the adequate sample size for both the patient population and the control group and the fact that our inclusion criteria for ARD patients minimize selection bias. However, this study has some limitations. First, it is a cross-sectional study with a retrospective design performed in a tertiary clinic (potential selection bias). Second, the information contained in the treatment history and disease activity of ARDs was not obtained from the electronic data system. We acknowledge that the observed association between ARDs and PHPT and the hypotheses put forward need to be further investigated for a definitive understanding.

## Conclusions

The prevalence of ARDs has increased in PHPT patients. The results of our study showed that PHPT accompanying ARDs developed after the development of rheumatologic diseases. The fact that PHPT developed during the course of ARDs suggests that the physiopathologic processes of ARDs may be a potential trigger for the development of PHPT. Localization efforts are less successful in PHPT associated with ARDs, a result that may be related to the higher incidence of multiglandular disease. Interacting mechanisms in ARDs seem to stimulate not only PTH but also parathyroid gland hyperplasia. This hypothesis needs to be supported by prospective studies.
